# Pathological Alterations and Stress Responses near DBS Electrodes after MRI Scans at 7.0T, 3.0T and 1.5T: An *In Vivo* Comparative Study

**DOI:** 10.1371/journal.pone.0101624

**Published:** 2014-07-02

**Authors:** Lin Shi, An-Chao Yang, Da-Wei Meng, Shao-Wu Li, Huan-Guang Liu, Jun-Ju Li, Xiu Wang, Xin Zhang, Jian-Guo Zhang

**Affiliations:** 1 Department of Functional Neurosurgery, Beijing Neurosurgical Institute, Capital Medical University, Beijing, China; 2 Department of Neurosurgery, Beijing Tiantan Hospital, Capital Medical University, Beijing, China; 3 Department of Neurosurgery, People's Hospital of Hainan Province, Haikou, Hainan Province, China; Oslo University Hospital, Norway

## Abstract

**Objective:**

The purpose of this study was to investigate the pathological alterations and the stress responses around deep brain stimulation (DBS) electrodes after magnetic resonance imaging (MRI) scans at 7.0T, 3.0T and 1.5T.

**Materials and Methods:**

DBS devices were stereotactically implanted into the brains of New Zealand rabbits, targeting the left nucleus ventralis posterior thalami, while on the right side, a puncture passage pointing to the same target was made. MRI scans at 7.0T, 3.0T and 1.5T were performed using transmit/receive head coils. The pathological alterations of the surrounding tissue were evaluated by hematoxylin and eosin staining (H&E staining) and transmission electron microscopy (TEM). The levels of the 70 kDa heat shock protein (HSP-70), Neuronal Nuclei (NeuN) and Caspase-3 were determined by western-blotting and quantitative polymerase chain reaction (QPCR) to assess the stress responses near the DBS electrodes.

**Results:**

H&E staining and TEM showed that the injury around the DBS electrodes was featured by a central puncture passage with gradually weakened injurious alterations. Comparisons of the injury across the groups manifested similar pathological alterations near the DBS electrodes in each group. Moreover, western-blotting and QPCR assay showed that the level of HSP-70 was not elevated by MRI scans (*p*>0.05), and the levels of NeuN and Caspase-3 were equal in each group, regardless of the field strengths applied (*p*>0.05).

**Conclusions:**

Based on these findings, it is reasonable to conclude that in this study the MRI scans at multiple levels failed to induce additional tissue injury around the DBS electrodes. These preliminary data furthered our understanding of MRI-related DBS heating and encouraged revisions of the current MRI guidelines for patients with DBS devices.

## Introduction

Deep brain stimulation (DBS) is a surging neurostimulation technique, and its effectiveness has been verified [1]. Because of the potential risks related to the heating of DBS leads in the radio frequency (RF) field of magnetic resonance imaging (MRI) [2]–[4], most patients with DBS devices choose CT rather than MRI for postoperative confirmation of the lead position [1], [5]. If MRI is irreplaceable, only 1.5T MRI with an RF of 64 MHz frequency is considered relatively safe when specific guidelines are followed [5]–[8]. In view of the former cases with terrible results induced by the MRI-related DBS heating, any violations from the guidelines are considered dangerous [2], [3], [9]. Non-1.5T MRI scans (i.e., 1.0T or 3.0T) are not recommended by Medtronic, the major DBS manufacturer [3], [9]. As indicated, however, the current recommendations concerning this issue, although proven to be protective and beneficial [7], [10], are considered too stringent for patients with DBS devices [11], because of the unfortunate inaccessibility to higher field MRI systems, which have a superior image quality [12]. Reassessment of the current guidelines related to the safety of patients with DBS devices in MRI scans may be required [5].

It has been suggested that the energy of heating predominately originates from RF resonance during MRI scanning [6], [13]. One dosimetric index used to evaluate the thermal effects of RF energy is the specific absorption rate (SAR), which is related to the RF intensity, the tissue properties, such as geometry and size, and the orientation of the exposed object in the RF field [14], [15]. The SAR value depends on the magnitude of the electric field and the properties of the absorbing object according to the following equation, ***SAR = σE^2^/2ρ***, where *σ* is the electrical conductivity of the tissue, *ρ* is the tissue density and *E* is the induced electrical field strength [16]–[18]. SAR has been used to evaluate the thermal effects of DBS devices in the RF field of MRI [19], [20]. It has been estimated that doubling the field strength from 1.5T to 3.0T leads to a quadrupling of the SAR [21], [22]. Thus, MRI scans at ultrahigh field strengths have been banned.

However, the evaluation of heating and its influences using SAR is problematic in patients with DBS devices. First, the calculation of SAR is over-simplified because it is substantially more complicated to estimate the actual electromagnetic field near a thin conductive implant (i.e., the DBS lead), which has electromagnetic properties that are significantly different from the surrounding tissue [18], [23], [24]. Second, a myriad of other factors influence the process, which makes it inaccurate to evaluate the thermal effects from the SAR value because it is hard to homogenize other factors in clinical practice [1], [16], [25], [26]. It is quite possible that similar SAR values with different experimental designs (i.e., difference in the orientation of the DBS devices, the length of the extension inside the coil and the parameters of the scan sequence) produce completely different heating results. Third, some investigations have compared the temperature elevations of DBS leads or other implants using MRI at different field strengths in the same phantoms or animals to minimize the disturbance of inhomogeneous experimental settings. These studies failed to detect the “expected” significant temperature elevation of DBS leads or implants in higher-field systems (i.e., 3.0T/128 MHz, 7.0T/300 MHz or higher), which contradicted the calculation results merely from the SAR using the aforementioned equation [27]–[31]. Other investigations have also compared the temperature alterations of DBS leads in approximately congruent settings except for different field strengths. These studies demonstrated lower temperature elevations in higher field systems [11], [13], [19], [32], [33], which discorded with the prediction that higher field systems induce greater heating than lower ones. Moreover, the actual in vivo conditions are much different from those in the phantom models. The inhomogeneity of tissular electric properties, the absorption of RF energy by the surface tissue and the heat dissipation facilitated by the blood flow et al., may all contribute to the reduction of the heating and its influences to the surrounding tissue during MRI scanning [13], [33]. It is possible that heating of the DBS leads detected in vitro can be mitigated or reduced by the natural heat dissipation mechanisms of the brain.

Given the above discussed, the MRI-related heating of DBS devices cannot be estimated merely by the SAR value. MRI scans at ultrahigh field strengths may not necessarily lead to immense heating of DBS devices in vivo. Thus, we designed this preliminary in vivo comparative study to test these hypotheses by comparing the tissular alterations near the DBS electrodes in response to the MRI scans at different field strengths.

## Materials and Methods

### Ethics Statements and Animal Grouping

This study was performed between 8 am and 3 pm. The study was in accordance with the recommendations from the Guidelines for the Use and Care of Experimental Animals and was approved by the Beijing Association on Laboratory Animal Care (Permit Number: SYXK 2010–0141). The entire surgery was conducted under urethane anesthesia (1 mg/kg, i.m., Sigma, St. Louis, MO, USA), and the average anesthesia time was approximately 1 hour. Atropine (0.5 mg/kg, i.m., Sigma, St. Louis, MO, USA) was used to inhibit the glandular secretions. The vital signs (heart rate: 100–120 bpm, respiration rate: 10–14/min, and temperature of the anus: 37.5–38.5 °C) were continuously monitored throughout the anesthesia. We maintained the temperature of the surgery room and the scan room at 24 °C. During MRI scanning, the rabbits' bodies were covered with a thick cloth to reduce heat dissipation. Every effort was made to minimize suffering during the procedure.

New Zealand rabbits were used because of the easy availability, the relative inferiority to primates, and a brain size that was sufficiently large for the DBS lead in comparison with rats. Forty-eight male adult rabbits (weighing 4.5–5.5 kg), provided by the Laboratory Animal Center of Military Medical Science Academy of China, were randomized to Group 1 (G1, 7.0T group, n = 12), Group 2 (G2, 3.0T group, n = 12), Group 3 (G3, 1.5T group, n = 12) and Group 4 (G4, control group, n = 12). Each group was evenly divided into two subgroups (A and B, n = 6) because further processing was different. According to previous studies, six animals per group were statistically sufficient to detect significant differences [34], [35]. The group information, which was completely blind to the experimenters, is shown in Table 1.

**Table 1:** Grouping information.

Groups	Subgroups	Procedures	MRI	Assays
G1	A(n = 6),	DBS(L)	7.0T	H&E/TEM
	B(n = 6)	DBS(L), P(R)	7.0T	WB/QPCR
G2	A(n = 6)	DBS(L)	3.0T	H&E/TEM
	B(n = 6)	DBS(L), P(R)	3.0T	WB/QPCR
G3	A(n = 6),	DBS(L)	1.5T	H&E/TEM
	B(n = 6)	DBS(L), P(R)	1.5T	WB/QPCR
G4	A(n = 6)	P(L)	7.0T	H&E/TEM
	B(n = 6)	*	7.0T	WB/QPCR

* Rabbits in group G4B underwent only general anesthesia for 2 hours without operation.

L: left. R: right. P: paracentesis. G: group. H&E staining: hematoxylin and eosin staining, TEM: transmission electron microscopy, HSP-70: the 70 kDa heat shock protein, QPCR: quantitative polymerase chain reaction.

### DBS Implantation

**Figure 1:**
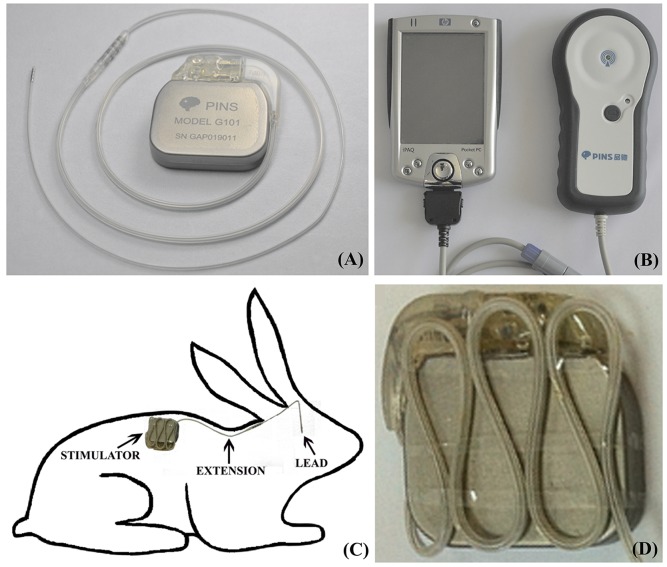
PINS DBS device and its placement in rabbits. (**A**) The PINS DBS device, Model G101, including the stimulator, extension and lead. (**B**) The controller and personal digital assistant for the PINS DBS device. (**C**) The stimulator of the PINS DBS device with zigzagged wire on its surface. (**D**) The placement of the PINS DBS device in rabbits.

The Programable Implantable Neurostimulation System (PINS) DBS system (Model G101, PINS Medical Co. Ltd., Beijing, China, Fig. 1) was used in this study. The effectiveness of this novel DBS system has been verified and was recently released for clinical use in China as a substitute for the Medtronic DBS devices [36]. The parameters of the PINS DBS system were close to those of the Medtronic system (detailed information regarding the PINS DBS system is shown in Table 2). The surgical operations were conducted by a team made up by a very experienced neurosurgeon who performed all the implantation procedures, two residents and an anesthetist, all from our institution. The left nucleus ventralis posterior thalami (VPN) were targeted using stereotactic devices for the rabbits (David KOPF Instruments, Tujunga, CA, USA) according to an atlas of the rabbit's brain (A Stereotaxic Atlas of the New Zealand Rabbit's Brain, Ivan Urban, Springfield, Charles C Thomas Publisher, 1972). A tailored guiding catheter (inside diameter: 2.1 mm) was used to create a passage during lead positioning. The VPN was chosen because its size and position were in favor of DBS implantation. The stereotactic coordinates of the VPN, which were verified in our pre-test, were 12.0 mm anterior to the posterior fontanel, 4.0 mm lateral to the sagittal suture, and 10.5 mm deep to the dura mater. The intracranial length of the lead was 14.0 mm. The extension was tunneled subcutaneously through the neck with the connector placed at the middle of the neck. The extra wire was not winded around the burr hole on the head, as some surgeons have performed in clinical practice. Instead, the extra wire was zigzagged on the surface of the stimulator (six to seven folds) with a sterilized band to reduce the wire located on the head (Fig. 1), which was assumed to be conducive in reducing the artifacts in MRI, as verified by our pre-tests. The stimulator was placed 8–10 cm below the last rib and 6–8 cm next to the spine. This positioning of the extension and the stimulator was designed to simulate the relative position and bearing of the DBS devices and the RF coil during the MRI examination. Paracentesis (puncture) to the same point on the right side of the experimental groups and on both sides of G4A was made by slowly withdrawing the DBS lead and catheter after insertion, thereby simulating the mechanical injury of the DBS implantation. The wounds were carefully sutured. The electrical impedance of the devices was examined to ensure the correct connection of the DBS devices, and the stimulators were turned off throughout the experiment. Rabbits of G4B underwent general anesthesia for 2 hours without operation.

**Table 2:** Measurements and settings of the PINS DBS system.

Measurements and Settings of the PINS DBS System			
Stimulator		Extension and Lead	
**Dimensions (mm)**	(47±2)×(52±2)×(11±1)	**Extension Length (mm)**	510±20
**Weight (g)**	35±3	**Extension Diameter (mm)**	2.5±0.2
**Stimulation Output**	Off	**Lead Length (mm)**	400±10
**Stimulation Mode**	Bipolar	**Lead Diameter (mm)**	1.3±0.2
**Amplitude (volt)**	0	**Electrodes Length (mm)**	1.5±0.1
**Impedance (Ω)**	>4000	**Electrodes Spacing (mm)**	0.5±0.1
**Other Parameters**	No Changes	**Distal Tip Distance (mm)**	0.5±0.1

### MRI Scans

**Figure 2:**
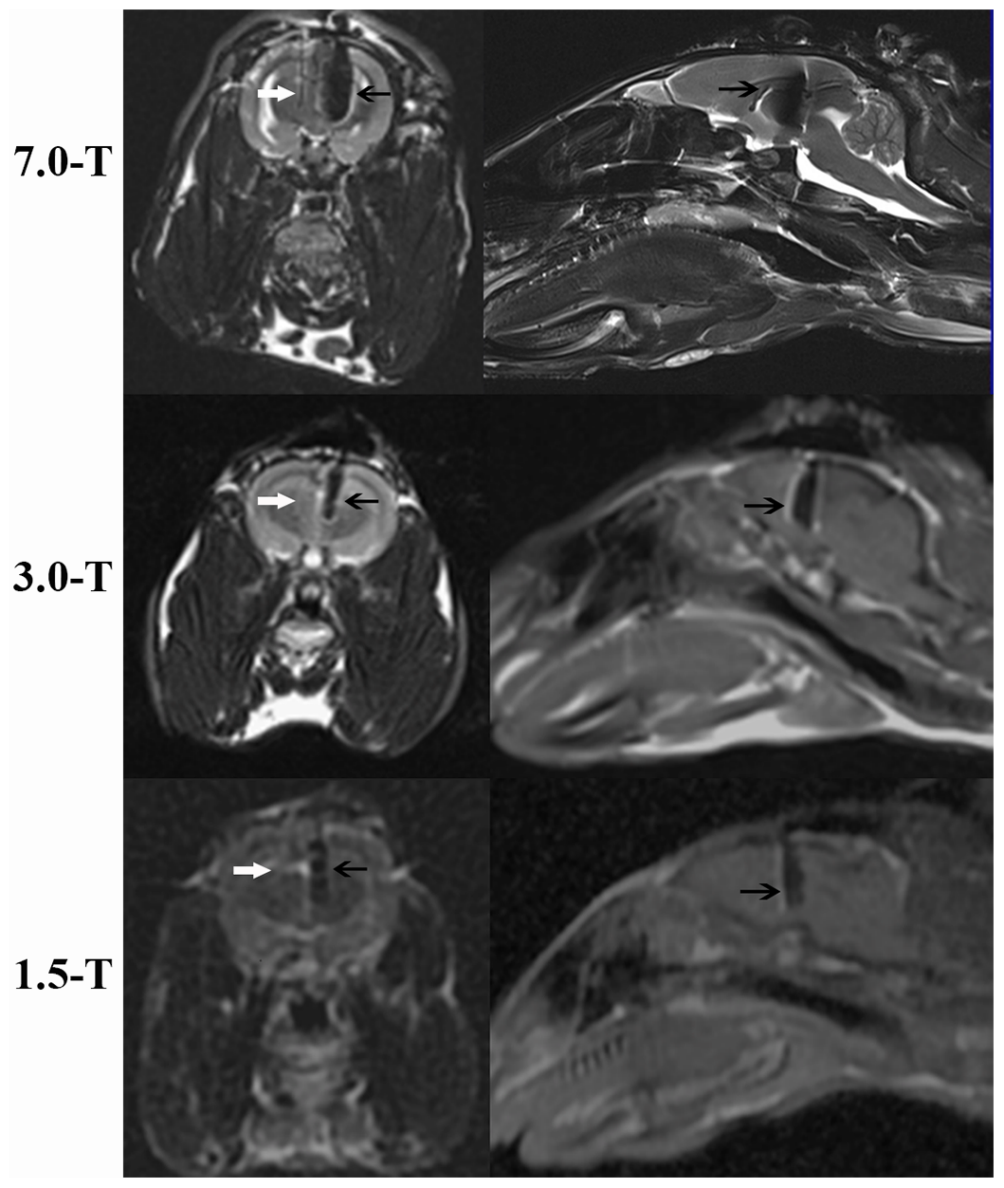
MRI (sagittal and axial sections) at levels of 7.0T, 3.0T and 1.5T. This image demonstrates the position of the lead (leftward, black arrow) that was covered by the artifact of the lead and the puncture passage (rightward, white arrow), which was unclear in the 3.0T and 1.5T images.

MRI scans were conducted at levels of 7.0T, 3.0T and 1.5T (1.5T: the Signa HDxt system, GE Healthcare, Little Chalfont, Buckinghamshire, UK; 3.0T: the Verio system, Siemens, Berkeley, CA, US; 7.0T: the ClinScan system, Bruker, Ettlingen, Germany) using transmit/receive coils (1.5T: GE Split head coil, part number 2336562; 3.0T: Siemens Matrix head coil, part number 7577832; 7.0T: Bruker Array head coil, part number 1059925), with the rabbits' heads secured within the head holders inside the RF coils. The rabbits were placed in a prone position with the head, neck and a small part of the body inside of the birdcage head coils. The rest of the body was outside of the coils, as well as the stimulator which was placed near the buttocks. Three separate runs composed of standard spin echo, gradient echo and fluid attenuated inversion recovery sequences were performed at each magnitude, which took more than 25 minutes. The required patient weight was entered as 50 kg so that the total emitted power would be close to that received by a 50 kg person in the actual scans. The scanner-displayed coil average SAR values were calculated from the entered patient weight and scan sequencies based on the modeling algorithms. In the actual scans, the predicted SAR should be the whole-head average SAR expected for a human weighing 50 kg, rather than a 50-kg animal with different anatomy. The displayed SAR values for each run were 0.24–2.20 W/kg for 1.5T MRI, 0.44–2.60 W/kg for 3.0T MRI and 0.52–2.96 W/kg for 7.0T MRI (Table 3). The location of the DBS leads was checked during the scanning. Only those rabbits with precise placement of the DBS lead in the MRI were included in the following experiments (Fig. 2). After MRI scanning, the rabbits were returned to their cages without feeding. Most rabbits awoke within 1 hour. No obvious neurological deficits were found.

**Table 3:** Information and Parameters of MRI Scanners.

Magnitude	Manufacturer	Scanner	Coil	Software	Runs	Time	SAR	Frequency
1.5T	GE	Signa HDxt	H, T/R	GE	3	32 min	0.52–2.96	64 MHz
3.0T	Siemens	Verio	H, T/R	Syngo	3	28 min	0.44–2.60	124 MHz
7.0T	Bruker	ClinScan	H, T/R	Syngo	3	25 min	0.24–2.20	300 MHz

H: head. T/R: transmit/receive. SAR: specific absorption rate.

### Pathology

**Figure 3:**
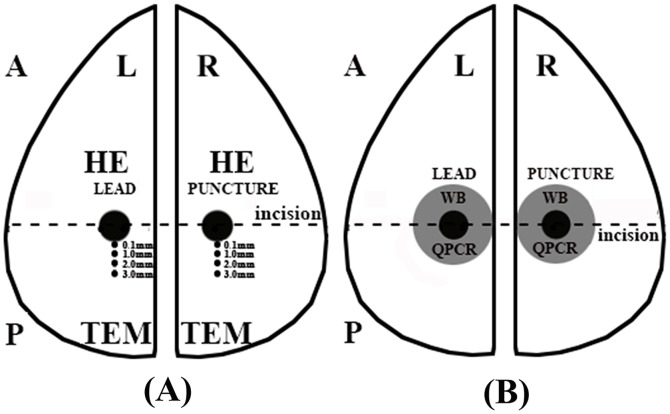
Processing of the rabbit brains for pathological and molecular examinations. The rabbit brains were dissected along the puncture passage on a coronal plane for pathological and molecular examinations. (A) For rabbits from the A subgroups, the anterior halves of the brains were processed for H&E staining and the posterior halves for TEM. (B) For rabbits from the B subgroups, the anterior halves of the brains were processed for western-blotting, and the posterior halves for QPCR. A: anterior. P: posterior. L: left. R: right. HE: H&E staining. TEM: transmission electron microscopy. WB: western-blotting. QPCR: quantitative reverse transcription polymerase chain reaction.

Twenty-four hours after MRI scanning, which is an appropriate time point to detect tissue injury and apoptosis [37], [38], the rabbits were euthanized and decapitated. The brains were dissected along the puncture passage on a plane vertical to the sagittal axis (Fig. 3 (A)). The samples near the end of the puncture passages from the anterior halves of the brains were processed for H&E staining. Observation was conducted using a light microscope (Axio imager A2, Carl Zeiss, Gottingen, Germany) at a distance of 0.1 mm, 1.0 mm, 2.0 mm and 3.0 mm away from the passage (as measured by the software of the microscope). At each distance, the overall injury was scored empirically in five random visual fields by two blinded pathologists using the criteria shown in Table 4. Tissue blocks of appropriate dimensions from the posterior halves of the brains were processed for TEM at a depth of 8 mm to the dura and at distances of 0.1 mm (immediately adjacent to the passage), 1.0 mm, 2.0 mm and 3.0 mm away from the passage. Ultrathin sections of the samples were observed and pictured using an electron microscope (H-7650 system, Hitachi, Chiyoda-ku, Tokyo, Japan). The ultrastructural changes were assessed empirically by two blinded pathologists from our institute. Neuron injury from each sample was scored in five random visual fields according to the criteria in Table 5. If there were several neurons in one visual field, the worst was scored. The scoring systems in Table 4 and Table 5 were designed by pathologists from Beijing Neurosurgical Institute based on clinical experiences and relevant references [39]–[44]. An increment of 0.5 points was added to the score when the injury was between two items in Table 4 and Table 5. When the scores from the two pathologists were the discordant, a third pathologist was called upon to help determine the final scores.

**Table 4:** The criteria for scoring tissue injury in H&E staining.

Scores	Observation
**0**	Normal. The cells and extracellular matrix were uninjured.
**1**	Mildly injured. Slightly swollen cells with decreased protrusions spread in the homogenous extracellular matrix. Pyknotic cells, apoptosis or necrosis was rarely observed.
**2**	Moderately injured. Oval, lightly stained swollen cells. Increased red neurons, pyknotic cells, apoptosis and necrosis were observed in the granulate matrix adorned with entangled fibers. Neuropils were swollen.
**3**	Severely injured. Red neurons, pyknotic cells and apoptotic cells were commonly observed. Eosinophilic ghost cells were interspersed in a structureless matrix with disorganized neuropils and tangling fibers.
**4**	Deadly injured. Eosinophilic ghost cells and conspicuous coagulative necrosis prevailed. Several pyknotic cells and red neurons interspersed in the cluttered matrix, which was filled with vacuoles left by dead neurons.

H&E staining: hematoxylin and eosin staining.

Minimal increment: 0.5 (Designed by Beijing Neurosurgical Institute, 2012).

**Table 5:** The criteria for scoring the neuronal injury in TEM.

Scores	Injurious manifestations of the neurons in TEM	
**0**	Basically normal.	
**1**	Slightly injured, focally distended ER, condensed or swollen M, fovea on karyolemma.	
**2**	Mildly injured, general swelling of organelles, clearly decreased cytoplasmic electron density, major depression of karyolemma.	
**3**	Moderately injured, severe swelling of the entire cell, formation of cytoplasmic vacuoles or blebs, evident cell shrinkage, transparent cytoplasm.	
**4**	Severely injured, pyknotic cells with deformed, but integrated cell membrane and karyolemma, apoptosis.	
**5**	Near death, pyknotic cells with trace of karyorrhexis or karyolysis, disruption of cell membrane, rupture of karyolemma, disintegration of organelles, apoptotic bodies.	

TEM: transmission electron microscopy. M: mitochondria. ER: endoplasmic reticulum.

Minimal increment: 0.5 (Designed by Beijing Neurosurgical Institute, 2012).

### Measurements of the 70 kDa Heat Shock Protein (HSP-70) and mRNA

Five hours after MRI scanning, when HSP-70 was at its peak [45], [46], the rabbits of the B subgroups were euthanized and decapitated. The brains were similarly dissected. The appropriate amount of tissue surrounding the end of the lead and the passage was processed for western-blotting and QPCR assay (Fig. 3 (B)). The HSP-70 quantity was determined by western-blotting using a mouse monoclonal antibody that specifically and exclusively recognizes the stress-induced species of HSP-70 (Abcam, Cambridge, MA, US). β-actin was used as a marker to quantify the relative quantity of HSP-70 in the cell. QPCR analysis was performed to quantify the HSP-70 mRNA. The sequence of the genes that encode HSP-70 was obtained on-line from the public NCBI database (Gene ID: 100352959). The following gene-specific DNA primers, which were designed to specifically recognize the mRNA of HSP-70 using Primer Premier version 5.0 (PREMIER Biosoft International, Palo Alto, CA, USA), were used on all rabbit tissue: forward, 5′- ATG GCC AAA GGC ACG GCG -3′; reverse, 5′- GCG GGT TCA GCG CCA CTG -3′. β-actin mRNA was used as a marker RNA to assess the relative quantity of HSP-70 mRNA: forward, 5′-TGA GAG GGA AAT CGT GCG TGA CAT-3′; reverse, 5′-ACC GCT CAT TGC CGA TAG TGA TGA-3′. The mRNA levels of HSP-70 and β-actin were relatively quantified using a fluorescence detection system (CFX96, BioRad, Hercules, CA, USA). We confirmed that each product made by the primers of the target gene showed a single melting curve.

### Measurements of Neuronal Nuclei (NeuN) and Caspase-3

Besides HSP-70, the levels of NeuN and Caspase-3 in the brain tissue around the DBS electrodes from G1B, G2B, G3B and G4B were also determined by western-blotting. The procedures of western-blotting were similar to those mentioned above. A mouse anti-NeuN monoclonal antibody (Millipore, Billerica, MA, US) and a rabbit anti-Caspase-3 polyclonal antibody (Abcam, Cambridge, MA, US) were employed. β-actin was used as a marker to make the protein levels standardized.

### Statistical Analysis

All quantitative data (i.e., H&E scores, TEM scores, protein levels and mRNA levels) were presented as the mean±SD (standard deviation) and analyzed statistically by one-way ANOVA analysis followed by an LSD (least significant difference) test using the SPSS 19.0 software program (IBM, New York, NY, US). In addition, Pearson's correlation test was used to examine the association between the HSP-70 levels and the SAR values. The difference was considered significant when *p*<0.05.

## Results

### Comparison of Pathological Alterations around the DBS Electrodes

H&E staining and TEM were carried out to reveal the microscopic and ultrastructural alterations induced by the DBS procedure and the potential heating effects around the DBS electrodes. The pathological alterations were compared across the groups at the same locations near the passages (i.e., 0.1 mm, 1.0 mm, 2.0 mm and 3.0 mm from the passages).

**Figure 4:**
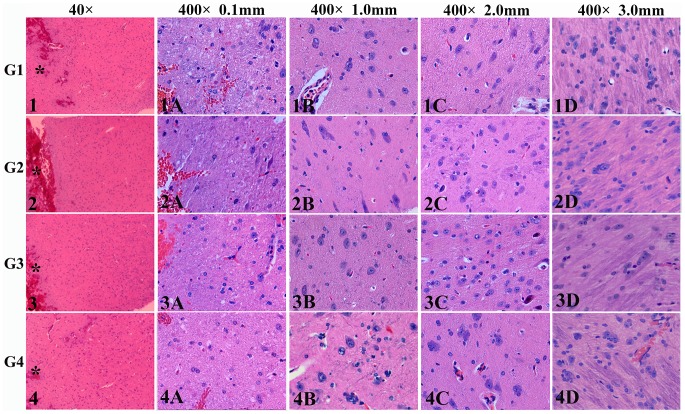
H&E staining showing the injury in the tissue around the DBS passages. (Panels 1, 2, 3 and 4, magnification ×40) The injurious alterations around the DBS electrodes was featured by a central puncture passage with injurious alterations that weaken with distance. (Panels 1A, 2A, 3A and 4A, 0.1 mm from the passages, magnification ×400) Severe tissue injury was manifested. Red neurons, ghost cells, vacuolization of the extracellular matrix, evident karyorrhexis, swelling cells and focal hemorrhage could be observed. (Panels 1B, 2B, 3B and 4B, 1.0 mm from the passages, magnification ×400) The overall tissue injury was less severe compared with 0.1 mm views. Red neurons, ghost cells, vacuolization of the extracellular matrix, evident karyorrhexis and swelling cells could still be observed, but less common. (Panels 1C, 2C, 3C and 4C, 2.0 mm from the passages, magnification ×400) The injury further relieved with much less apoptosis and red neurons. Swelling of the cells was still prevalent but less severe. (Panels 1D, 2D, 3D and 4D, 3.0 mm from the passages, magnification ×400) The injury became inconspicuous. Some cells were only slightly swelling. Rough comparisons of the pathology across the groups failed to reveal distinctive differences among G1, G2, G3 and G4 in terms of the injury extent and scope. *: DBS passage.

**Figure 5:**
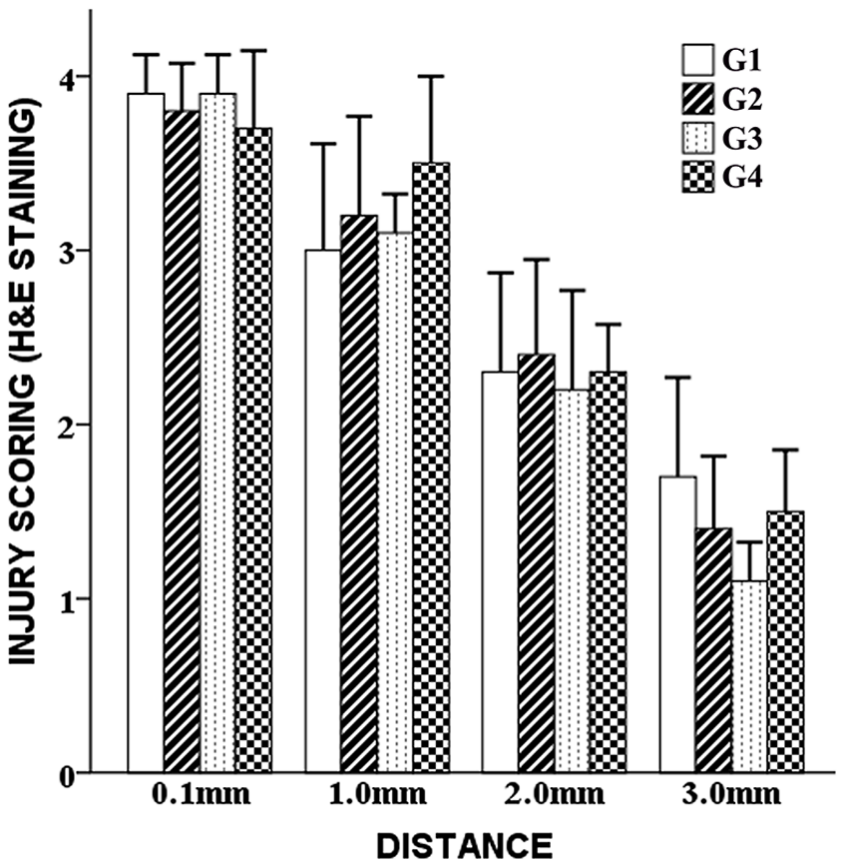
Injury scoring of H&E staining. Semi-quantification of H&E staining revealed that at each location the differences of the injury scores among G1, G2, G3 and G4 were not significant (p>0.05).

**H&E Staining. Observations**: as shown by H&E staining (Fig. 4, Panels 1, 2, 3, 4, Magnification ×40), the pathology around the DBS leads was featured by a central puncture passage with injurious alterations that gradually weakened with distance. In the immediate vicinity of the passages (0.1 mm from the passage, measured by the tools of the microscope) (Fig. 4, Panels 1A, 2A, 3A, 4A, Magnification ×400), severe injury was observed. Red neurons, ghost cells and vacuolization of the extracellular matrix were obvious and prevalent. Karyorrhexis and focal hemorrhage could be frequently observed. The remaining cells were either severely swelling or evidently pyknotic. At 1.0 mm away from the passages (Fig. 4, Panels 1B, 2B, 3B, 4B, Magnification ×400), the level of the injury decreased remarkably. Pyknotic cells, swelling cells, red neurons and apoptosis were still abundant. The background matrix was less sparse. At 2.0 mm away from the passages (Fig. 4, Panels 1C, 2C, 3C, 4C, Magnification ×400), a further decline of the injurious extent could be observed. Oval, lightly stained and mildly swelling cells were observed in the granulated extracellular matrix. Apoptosis, pyknotic cells and red neurons were much less common. Ghost cells or background vacuolization were not observed. At 3.0 mm away from the passages (Fig. 4, Panels 1D, 2D, 3D, 4D, Magnification ×400), the injury became very dim. Neurons with darkly stained Nissl's bodies and clear projections were abundant. Apoptosis and pyknotic cells almost disappeared. Slightly swelling cells could rarely be observed. The extracellular matrix was compact. Rough comparisons of the pathological alterations failed to reveal significant differences among G1, G2, G3 and G4 in terms of the injurious extent and scope, since identical pathological alterations were observed at each location. **Scoring**: the injury scores of H&E staining were presented as follows. G1: 3.900±0.224 (0.1mm), 3.000±0.612 (1.0mm), 2.300±0.570 (2.0 mm), 1.700±0.570 (3.0 mm). G2: 3.000±0.612 (0.1 mm), 3.200±0.570 (1.0 mm), 3.100±0.224 (2.0 mm), 3.500±0.570 (3.0 mm). G3: 2.300±0.570 (0.1 mm), 2.400±0.548 (1.0 mm), 2.200±0.570 (2.0 mm), 2.300±0.274 (3.0 mm). G4: 1.700±0.570 (0.1 mm), 1.400±0.418 (1.0 mm), 1.300±0.447 (2.0 mm), 1.500±0.354 (3.0 mm). ANOVA and LSD test revealed that at each location the differences of the injury scores among G1, G2, G3 and G4 were not significant (*p* = 0.133–0.759, *p*>0.05) (Fig. 5).

#### Transmission Electron Microscopy

**Figure 6:**
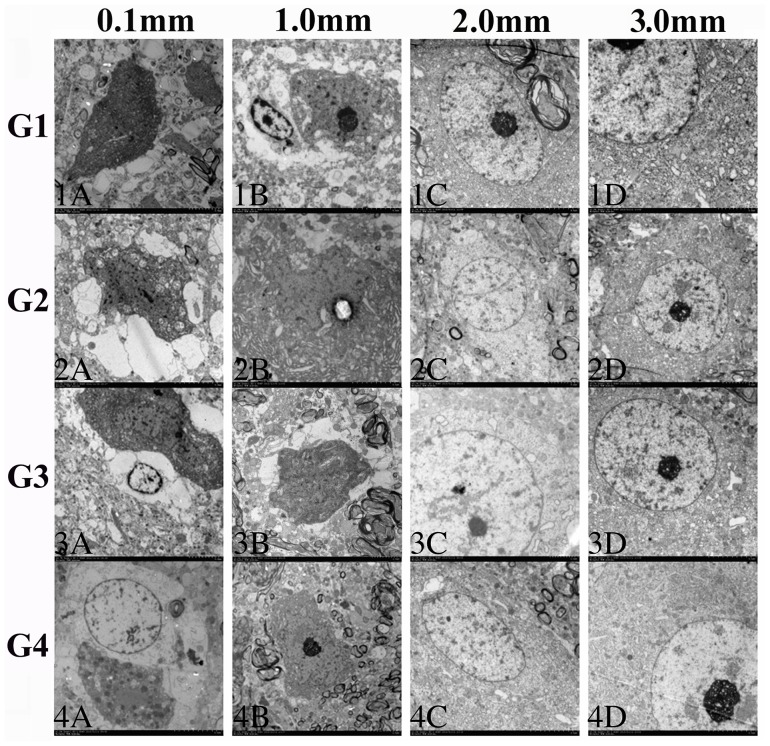
TEM showing the ultrastructural alterations in the tissue around the DBS passage. (Panels 1A, 2A, 3A and 4A, 0.1 mm from the passages, magnification ×1000) Severe tissue injury could be observed. Cracked fragments from disintegrated organelles, pyknotic neurons with incomplete cell membrane, neurons with deformed or ruptured nuclei, karyorrhexis, karyolysis and overwhelmingly swelling astrocytes and oligodendrocytes could be observed. (Panels 1B, 2B, 3B and 4B, 1.0 mm from the passages, magnification × 1000) The injury declined slightly. Remarkably pyknotic cells and severely swelling glial cells could be observed. (Panels 1C, 2C, 3C and 4C, magnification × 900–1000) The injury weakened greatly. Swelling of the neurons and glial cells became inconspicuous. In some neurons, only a proportion of the endoplasmic reticulum and mitochondria were swelling. (Panels 1D, 2D, 3D and 4D, magnification ×900–1000) The injury was substantially alleviated compared with the alterations of the former views. Some parts of the samples at this location could hardly be distinguished from the normal tissue. Rough comparisons of the ultrastructural alterations failed to reveal distinctive differences among G1, G2, G3 and G4.

**Figure 7:**
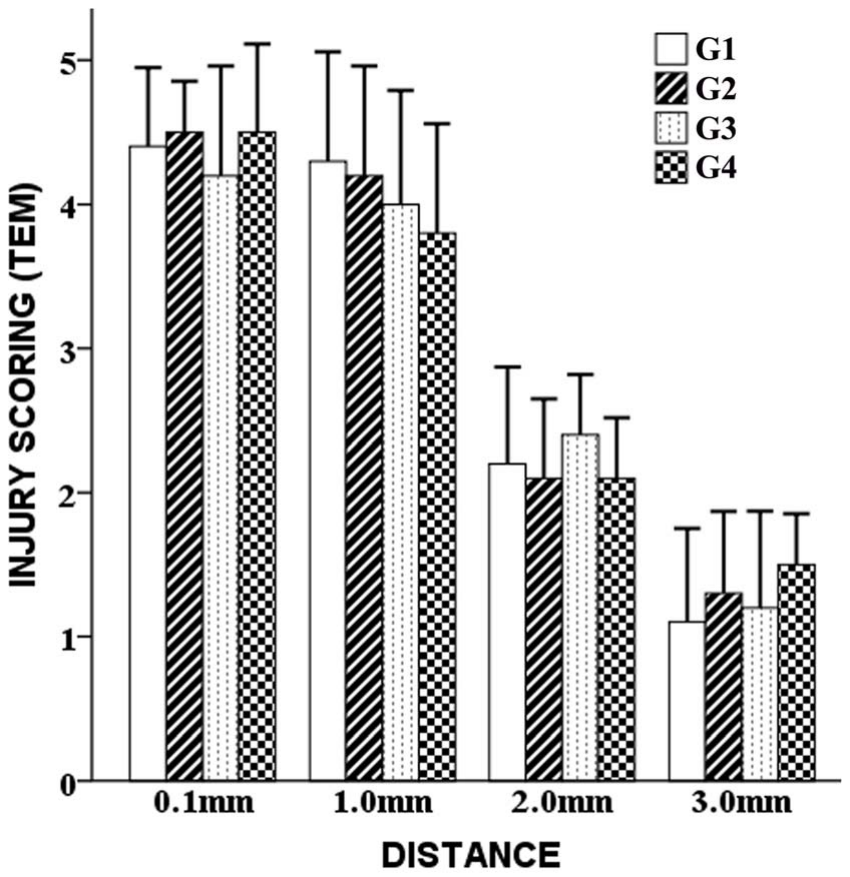
Injury scoring of TEM. Semi-quantification of TEM revealed that at each location the differences of the injury scores among G1, G2, G3 and G4 were not significant (p>0.05).

**Observations**: as shown by TEM images (Fig. 6, the images focused mainly on the ultramicroscopic alterations of neurons), the overall injury of the four groups was assessed at locations 0.1 mm, 1.0 mm, 2.0 mm and 3.0 mm away from the DBS passages. At 0.1 mm (Fig. 6, Panels 1A, 2A, 3A, 4A, Magnification ×1000), the overall injury was very severe. Large amounts of cracked fragments from disintegrated organelles were observed. The neurons were predominantly pyknotic, some with a complete cell membrane and some without. The nuclei were either deformed or ruptured. Karyorrhexis and karyolysis were common. The glial cells (astrocytes and oligodendrocytes) were overwhelmingly swelling, some of which were disintegrated. Cavitated axons with a loosened myelin sheath were frequently observed. The structure of the blood brain barrier was usually incomplete, with huge foot processes of astrocytes and a ruptured endothelium. At 1.0 mm (Fig. 6, Panels 1B, 2B, 3B, 4B. Magnification ×1000), the injury declined slightly. Although many cells remained remarkably pyknotic, the nuclei were usually deformed but not disintegrated. The quantity of crushed cells decreased, as well as the released fragments of cracked organelles. The glial cells remained severely swelling, although a bit better compared with the situations at 0.1 mm. Some axons were swelling with looming neurofilaments. At 2.0 mm (Fig. 6, Panels 1C, 2C, 3C, 4C. Magnification ×900–1000), the injury was greatly relieved because less pyknosis was observed. Swelling of the neurons and glial cells were diminished. In some neurons, only a proportion of the endoplasmic reticulum and mitochondria were swelling, and in some astrocytes, only decreased electron density was observed. The myelin sheath remained evidently swelling. The capillaries were much less tumid with deformed, stenotic but larger lumens. At 3.0 mm (Fig. 6, Panels 1D, 2D, 3D, 4D. Magnification ×900–1000), the injury was substantially alleviated compared with the situations in the former locations. The organelle swelling in some neurons and glial cells was inconspicuous. Layer-like separation of axons was less evident. The stenosis and deformation of capillaries were slight. Some parts of the samples at this location could hardly be distinguished from the normal tissue. Generally, the injury declined with distance in each group. Rough comparisons failed to reveal distinctive differences among G1, G2, G3 and G4 in terms of the ultrastructural alterations, since the injury extent of the neurons and glial cells was similar in these groups. **Scoring**: the injury scores of TEM were presented as follows. G1: 4.400±0.547 (0.1 mm), 4.300±0.758 (1.0 mm), 2.200±0.671 (2.0 mm), 1.100±0.652 (3.0 mm). G2: 4.500±0.354 (0.1 mm), 4.200±0.758 (1.0 mm), 2.100±0.548 (2.0 mm), 1.300±0.570 (3.0 mm). G3: 4.200±0.758 (0.1 mm), 4.000±0.791 (1.0 mm), 2.400±0.418 (2.0 mm), 1.200±0.671 (3.0 mm). G4: 4.500±0.612 (0.1 mm), 3.800±0.758 (1.0 mm), 2.100±0.418 (2.0 mm), 1.500±0.354 (3.0 mm). ANOVA and LSD test revealed that at each location the differences of the TEM scores among G1, G2, G3 and G4 were not significant (*p* = 0.087–0.653, *p>0.05*) (Fig. 7).

### Stress Responses in the Tissue surrounding the Passages

HSP-70, NeuN and Caspase-3 were considered to be indices for stress responses. Therefore, western-blotting and QPCR on these proteins were performed to detect and compare the insults imposed on cells induced by the DBS procedures and the potential heating effects triggered by MRI scans.

#### Western-blotting on HSP-70 Protein

**Figure 8:**
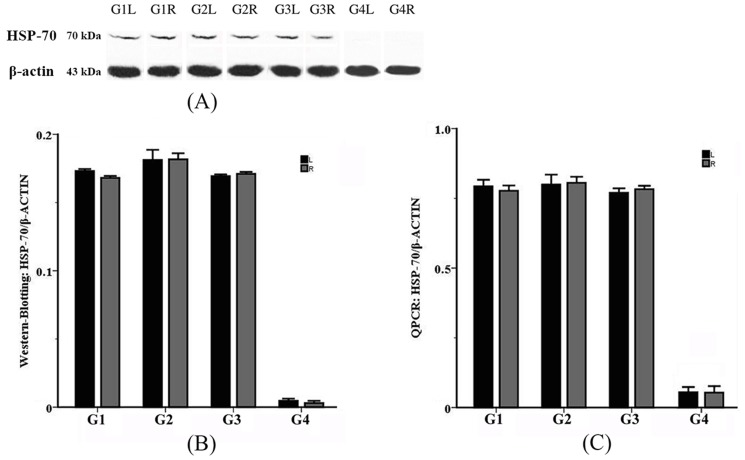
Western-blotting and QPCR assay of HSP-70 expression around the DBS passages. HSP-70 expression around the DBS passages was examined by western-blotting and QPCR assay. (A) A representative photo showing the protein bands of HSP-70 was presented. (B) Western-blotting assay showed that the differences of HSP-70 protein levels were not significant either between the left and the right sides in each group (p>0.05), or among G1, G2 and G3 (p>0.05). The HSP-70 levels in the experimental groups (G1, G2, G3) was significantly higher than that of the control group (G4) (p<0.05). (C) QPCR assay of HSP-70 mRNA in G1, G2, G3 and G4 showed that the differences of HSP-70 mRNA were not significant either between the left and the right sides in each group (p>0.05), or among G1, G2 and G3 (p>0.05). The HSP-70 expression in the experimental groups (G1, G2, G3) was significantly higher than that of the control group (G4) (p<0.05).

A representative photo showing the protein bands is presented in Fig. 8 (A). After normalization to β-actin, the relative levels of HSP-70 in each group were 0.173±0.014 (G1L), 0.168±0.014 (G1R), 0.181±0.075 (G2L), 0.182±0.044 (G2R), 0.169±0.012 (G3L), 0.171±0.015 (G3R), 0.005±0.002 (G4L), and 0.003±0.001 (G4R). Statistical process with one-way ANOVA and LSD tests revealed that the differences of HSP-70 protein levels were not significant either between the left and the right sides in each group (*p* = 0.522-0.692, *p*>0.05), or among G1, G2 and G3 (*p* = 0.398–0.430, *p*>0.05). The HSP-70 levels in the experimental groups (G1, G2, G3) was significantly higher than that of the control group (G4) (*p* = 0.000, *p*<0.05). (Fig. 8 (B)). Besides, no correlation between the levels of HSP-70 and the applied SAR values was found by Pearson's correlation test (*p* = 0.901, *p*>0.05).

#### QPCR on HSP-70 mRNA

The results are presented in a bar graph shown in Fig. 8 (C). After normalization to β-actin, the relative levels of HSP-70 mRNA were 0.793±0.024 (G1L), 0.777±0.019 (G1R), 0.799±0.036 (G2L), 0.806±0.022 (G2R), 0.770±0.016 (G3L), 0.783±0.012 (G3R), 0.054±0.020 (G4L), and 0.049±0.025 (G4R). HSP-70 mRNA expression was significantly higher in the experimental groups compared with the control group (*p* = 0.000–0.032, *p<0.05*). However, no significant difference was found between the left side and the right side in each group (*p* = 0.113–0.696, *p>0.05*).

### Western-blotting on NeuN and Caspase-3

**Figure 9:**
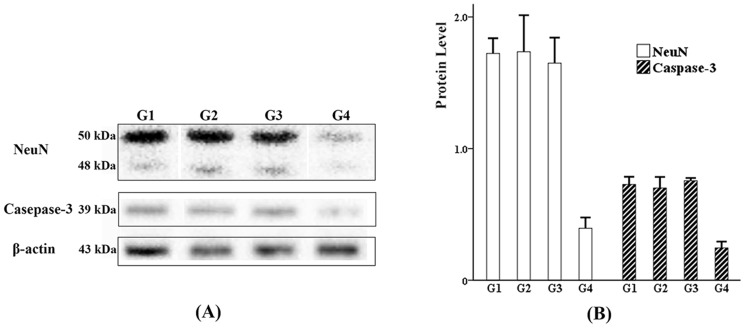
Western-blotting of NeuN and Caspase-3 in the tissue around the DBS passages. (A) A representative photo showing the protein bands of NeuN and Caspase-3 was presented. (B) Statistical process revealed that the differences in NeuN and Caspase-3 levels were not significant among G1, G2 and G3 (p>0.05), whereas the levels of the two stress proteins in the experimental groups (G1, G2, G3) were significantly higher than those of the control group (G4) (p<0.05).

A representative photo showing the protein bands is presented in Fig. 9 (A). After normalization to β-actin, the relative levels of NeuN in each group were 1.723±0.115 (G1), 1.735±0.278 (G2), 1.649±0.194 (G3) and 0.395±0.082 (G4). The relative levels of Caspase-3 in each group were 0.728±0.055 (G1), 0.701±0.079 (G2), 0.756±0.019 (G3) and 0.245±0.046 (G4). Statistical process with one-way ANOVA and LSD tests revealed that the differences in NeuN and Caspase-3 levels were not significant among G1, G2 and G3 (*p* = 0.326-0.890, *p*>0.05), whereas the levels of the two stress proteins in the experimental groups (G1, G2, G3) were significantly higher than those of the control group (G4) (*p* = 0.000, *p*<0.05) (Fig. 9 (B)).

## Discussion

In the present study, we investigated and compared the tissular alterations near the DBS electrodes in response to the MRI scans at different field strengths. H&E staining and TEM showed that the injury was severe with obvious necrosis in the vicinity of the passage (i.e., 0.1 mm and 1.0 mm) and gradually diminished with distance (i.e., 2.0 mm and 3.0 mm). The injury near the DBS leads originated from two sources: the injury caused by the DBS implantation and the potential heating injury from the RF power. Considering that the rabbit brains are much smaller in size than the human brains, it is understandable that implantation of DBS leads designed for human may induce relatively greater injury on rabbits than on humans, which is considered why the influenced area was larger than the DBS passages. Another reason for the disaccordance between the injury scope and the size of the DBS passage is that the catheter (the inside diameter was 2.1 mm) employed in the study was removed after implantation of the DBS lead. In addition, we compared the pathology across the groups to find out whether the injury in ultrahigh MRI groups was more severe. To ensure that the comparisons were performed more objectively, the morphological findings were semi-quantified based on our scoring systems. Morphological observations showed that the injury of each group was similar, and quantification of the injury failed to reveal significant differences among the groups, indicating that MRI scans at multiple field strengths failed to induce morphologically detectable injury near the DBS electrodes.

Moreover, we performed molecular examinations to evaluate the injury around the DBS leads. Inducible HSP-70 has been used as a marker to estimate the overall damage to cells [47], [48], and the elevation of inducible HSP-70 parallels the total injury to cells [47], [49], even when the injury is inflicted in two steps [50]. Thus, we determined the quantity of inducible HSP-70 to study whether there were differences between the two sides in terms of the total injury. The results from repeated western-blotting and QPCR experiments failed to show significantly higher levels of HSP-70 compared with the contralateral sides (*p*>0.05), and no correlation between the levels of HSP-70 and the applied SAR values was found. These findings implied that the levels of HSP-70 were not influenced by the MRI scans and the SAR values. Moreover, we determined other proteins that are considered as indices for stress responses, i.e., NeuN and Caspase-3 [51], [52]. Results showed that the levels of NeuN and Caspase-3 were equal in each group, suggesting that the stress responses triggered by MRI scans were not related the MRI field strength.

Taken together, these results strongly implied that in this study MRI scans at ultralhigh field strengths failed to induce additional heating injury near the DBS electrodes. Although it is hard to say whether our findings apply to other occasions with different settings, our data at least supports the idea that ultrahigh field MRI scan can be safely performed in certain circumstances. Coincidentally, identical results were observed by an on-going investigation in our institute that study the behavioral changes after MRI scans at multiple field strengths using a robust number of Rhesus monkeys (not published). Some studies have demonstrated 1.5T MRI as relatively safe for patients with DBS devices.

The reasons why MRI at ultrahigh levels with more powerful RF energy failed to induce greater heat on the DBS leads, we assume, might lie in the following aspects. First, in this study standard MRI scans with relatively low SAR values were applied, with the parallel placement of the extra-cranial part of the extension and the transmit/receive birdcage head coils outside which the RF field weakens sharply. These were all factors that tend to minimize the heating effects of the DBS devices. Moreover, the penetration of RF energy may be another reason. It has been proven that the absorption of RF energy at a very high frequency (>10 GHz) primarily occurred at the surface [53]. Yan Liu et al. [33] calculated the local SAR near the implants within a certain depth of tissue in the RF field of the MRI based on their computation formulas. The results showed that the SAR value of the 1.5T/64 MHz system was higher compared with the 3.0T/128 MHz system. The author assumed that this might be because the loss of RF energy was more obvious in higher field conditions in the tissue. Another study conducted by Muranaka et al. [17] with 1.5T/64 MHz and 3.0T/128 MHz systems showed that RF attenuated faster in the tissue at a higher frequency. Syed et al. [13] also constructed a mathematical model to assess the SAR value and temperature elevation of DBS electrodes in the 1.5T/64 MHz and 3.0T/128 MHz RF fields. The results in the 3.0T/128 MHz system were substantially lower, which further supported this assumption. Although no general conclusions could be reached based on these studies, it is reasonable to assume that under certain circumstances the RF radiation of MRI scanning at ultrahigh fields passes more energy superficially than deeply, which might be another possible explanation of our results.

Our findings have clinical significance because this study is the first attempt to investigate and compare the heating of DBS leads in MRI scans at multiple field strengths and its influences to the surrounding tissue. It is also the first multi-animal experiment that provides in vivo data concerning the safety of DBS devices during MRI scanning. Although substantial differences exist between humans and rabbits, the overall heating process is similar since the major factors that influence the heating effects have been made to simulate the actual scans performed on humans, including the scan sequences, parameters, dimensions and relative bearings of DBS devices, et al. Another advantage of our study is that the relatively smaller body mass of the rabbits tends to magnify the supposed heating effects because the coil average SAR value would be much higher in smaller animals with smaller body weights than in larger animals. Therefore, the finding that no heating injury was detected in the study provides us more confidence when conducting MRI scans on larger ones. Although the parameters applied in our study might be within the safety limits that would not trigger huge heat production, we at least demonstrated that ultrahigh field MRI (3.0T and 7.0T) could be performed safely with DBS devices implanted in the living body under certain conditions. Thus, our results support the idea that the heating of DBS devices in standard MRI scans is not a formidable issue if certain rules are followed. As proposed by Dr. Tagliati and Dr. Larson, current in vivo data have suggested a favorable risk/benefit ratio for patients with DBS to perform brain MRI scans when certain precautions are followed. More in-depth studies must be conducted, but our study strongly reminds us of the possibility that heating of DBS devices by MRI scans might not be as serious as previously anticipated. Perhaps it is time to consider the revision of the current guidelines for the use of MRI in patients with DBS devices [5], [7].

This in vivo study is preliminary and has limitations, primarily regarding its use of rabbits. Substantial anatomical discrepancies in shape and dimensions between humans and rabbits make the findings of this study less meaningful to clinical practice. Nevertheless, as described previously, the primary goal of our study was to compare the responses of the tissue surrounding the DBS leads and to identify possible differences in the injury scope and extent in the same experimental settings. Therefore, our results have meaning in that they showed that ultrahigh systems can induce similar changes as with 1.5T. Our data might also have significance to infant patients with implanted neurostimulation devices, as many infants are implanted with vagus nerve stimulation devices to treat epilepsy. Despite the disadvantages, the easy availability and the relative inferiority of rabbits to primates support the suitability of our approach for a preliminary study.

Our study is further limited because no direct temperature data were presented. The thermometer that measures the temperature changes of the DBS devices was currently inaccessible due to financial reasons. Besides, our study paid more attention to the morphological and molecular alterations in response to the heating of the DBS leads induced by MRI scans. The significance of our study was to provide preliminary in vivo data concerning the tissue responses to DBS heating. Other limitations of this study involved the use of general anesthetics during MRI scanning. It is well known that anesthetic agents disrupt cerebral blood flow and reduce the baseline brain temperature [54], [55]. Thus, the influence on the heating of the DBS leads was unpredictable. We monitored and maintained the vital signs at a stable level and kept the room temperature at 35 °C to minimize the influence of general anesthesia. Besides, as previous investigations have indicated, the temperature at the DBS electrodes was primarily dominated by the heating of the DBS leads because of the RF radiation and not by the relatively slow changes of the brain metabolism induced by anesthesia [11]. Moreover, our data may also be useful to the sedated or unconscious patients because the metabolism of the brain brains is similar [56].

Because of these limitations, the data acquired by our study are insufficient to draw generalized conclusions regarding the safety of patients with implants during MRI scanning at ultrahigh field strength. Thus, more in-depth studies are required to study the MRI-induced DBS heating in more universal conditions, i.e., using other placements of the DBS extension and other DBS devices with different dimensions, using larger animals, et al.

## Conclusions

To our knowledge, this preliminary investigation is the first randomized controlled multi-animal study assessing the heating of DBS leads during MRI scanning as well as being the first in vivo study that compared the pathological and molecular responses of the brain tissue surrounding the DBS leads in MRI scans at 7.0T, 3.0T and 1.5T field strengths. The results showed that MRI scans at ultrahigh field strengths failed to induce morphologically detectable injury on the surrounding tissue. Moreover, the examinations of HSP-70, NeuN and Caspase-3 suggested that MRI scans failed to trigger additional stress responses near the DBS electrodes. Based on these findings, we conclude that in this study the MRI scans at ultrahigh field strengths failed to induce evident heating injury around the DBS electrodes. Although it is inappropriate to generalize the conclusions to other experiments in different settings, these preliminary data are encouraging regarding the future use of 3.0T and 7.0T MRI in patients with implanted neurostimulation devices.
